# Prevalence of pulmonary TB and spoligotype pattern of *Mycobacterium tuberculosis *among TB suspects in a rural community in Southwest Ethiopia

**DOI:** 10.1186/1471-2334-12-54

**Published:** 2012-03-13

**Authors:** Amare Deribew, Gemeda Abebe, Ludwig Apers, Alemseged Abdissa, Fetene Deribe, Kifle Woldemichael, Chali Jira, Markos Tesfaye, Jafar Shiffa, Abraham Aseffa, Mesele Bezabih, Tadiye Abeje, Robert Colebunders

**Affiliations:** 1 Department of Epidemiology, Jimma University, Jimma, Ethiopia; 2 University of Antwerp, Antwerp, Belgium; 3 Department of Laboratory Sciences and pathology, Jimma University, Jimma, Ethiopia; 4 Institute of Tropical Medicine, Antwerp, Belgium; 5 Department of Health service management and Planning, Jimma University, Jimma, Ethiopia; 6 Department of psychiatry, Jimma University, Jimma, Ethiopia; 7 Department of Internal Medicine, Jimma University, Jimma, Ethiopia; 8 Armour Hansen Research Institute, Addis Ababa, Ethiopia

**Keywords:** Pulmonary TB, Prevalence, Spoligotype, Southwest Ethiopia

## Abstract

**Background:**

In Ethiopia where there is no strong surveillance system and state of the art diagnostic facilities are limited, the real burden of tuberculosis (TB) is not well known. We conducted a community based survey to estimate the prevalence of pulmonary TB and spoligotype pattern of the *Mycobacterium tuberculosis *isolates in Southwest Ethiopia.

**Methods:**

A total of 30040 adults in 10882 households were screened for pulmonary TB in Gilgel Gibe field research centre in Southwest Ethiopia. A total of 482 TB suspects were identified and smear microscopy and culture was done for 428 TB suspects. Counseling and testing for HIV/AIDS was done for all TB suspects. Spoligotyping was done to characterize the *Mycobacterium tuberculosis *isolates.

**Results:**

Majority of the TB suspects were females (60.7%) and non-literates (83.6%). Using smear microscopy, a total of 5 new and 4 old cases of pulmonary TB cases were identified making the prevalence of TB 30 per 100,000. However, using the culture method, we identified 17 new cases with a prevalence of 76.1 per 100,000. There were 4.3 undiagnosed pulmonary TB cases for every TB case who was diagnosed through the passive case detection mechanism in the health facility. Eleven isolates (64.7%) belonged to the six previously known spoligotypes: T, Haarlem and Central-Asian (CAS). Six new spoligotype patterns of *Mycobacterium tuberculosis*, not present in the international database (SpolDB4) were identified. None of the rural residents was HIV infected and only 5 (5.5%) of the urban TB suspects were positive for HIV.

**Conclusion:**

The prevalence of TB in the rural community of Southwest Ethiopia is low. There are large numbers of undiagnosed TB cases in the community. However, the number of sputum smear-positive cases was very low and therefore the risk of transmitting the infection to others may be limited. Active case finding through health extension workers in the community can improve the low case detection rate in Ethiopia. A large scale study on the genotyping of *Mycobacterium tuberculosis *in Ethiopia is crucial to understand transmission dynamics, identification of drug resistant strains and design preventive strategies.

## Background

Tuberculosis (TB) is one of the most challenging communicable diseases for developing countries particularly in Sub-Saharan Africa. A total of 8.8 million incident TB cases have been registered in 96 countries in 2010 [1]. The spread of this disease is fuelled by several factors notably the HIV/AIDS epidemic, low socio-economic status, overcrowding and malnutrition [2-4].

African countries south of the Sahara including Ethiopia are heavily affected by TB. The World Health Organization (WHO) global reports on TB showed that Ethiopia is among the ten top high burden countries in terms of prevalence or incidence cases of TB [5,6].

However, the real burden of TB in Ethiopia is not known due to several reasons. First, there is neither a reliable disease notification system, nor has any regular nation-wide epidemiological survey of TB and HIV been conducted. Second, Ethiopia has poorly developed diagnostic facilities and low health service coverage which might contribute for the very low case detection rate [7]. In the absence of a strong surveillance system, state of the art diagnostic laboratories and high health service coverage, community-based prevalence surveys are good alternatives to estimate the burden of TB and to evaluate the impact of the TB control program. We performed such a survey in a predominantly rural area of Southwest Ethiopia to estimate the prevalence of TB and to characterize the *Mycobacterium tuberculosis *strains circulating in the community.

## Methods

### Study area and study population

This cross sectional survey was conducted from February to March 2009 in the Gilgel Gibe field research centre, located in Jimma Zone about 260 km Southwest of Addis Ababa around the reservoir of the Gilgel Gibe hydroelectric dam. The site is bounded by four districts (Woredas): Sekoro, Omo-Nada, Tiro- Afeta and Kersa. In the four Woredas, two urban and eight rural Kebeles (smallest administrative units), were selected as field research center by Jimma University in 2005. They are found within 10 km of the reservoir of the dam. Since 2005, demographic and AIDS mortality surveillances has been undertaken by Jimma University in the research center. The total population of the field research center is 50156 with the total households of 10,882. The study population consisted of adults above 14 years of age and TB suspects in the study area.

### Sample size calculation and data collection procedures

The sample size was calculated using the following assumptions: prevalence of TB in rural community of 0.36% [6], 95% CI, margin of error of 20% and non-response rate of 10%. This gave us a total sample of 28917. Since the total adult population in Gilgel Gibe field research centre was 30040 (almost equal to the sample size), all adults > = 15 years were screened for TB.

A one-page screening questionnaire was prepared in Amharic (local language) to identify all adult TB suspects in the study area. Trained skilled workers who had completed high school education visited each household. If the heads of the households were not available during the visit, the data collectors repeatedly visited the same household 3 times. All adult (age > = 15 years) with a cough of two weeks or more were considered as TB suspect and given a sputum cup to bring a morning sputum to the nearby school or clinic (appointment centre) the next day. TB suspects were also asked to provide a second "on the spot" sputum sample during their visit to the appointment centre. During their visit, the TB suspects were interviewed about the presence of other symptoms of TB such as fever, their socio-demographic characteristics and perception towards TB using a structured questionnaire. We asked the presence of fever using Affan Oromo (the local language). The study area is malaria endemic and fever is well-known by the local language. Every TB suspect was counseled and asked consent to be tested for HIV. Screening for HIV was done using the KHB test (Shanghai Kehua Bio-Engineering Ltd, Shanghai, China; 2008). A positive sample was retested using the STAT-PAK test (Chembio Diagnostic System Inc, Medford, NY, USA; 2008). Collected sputum samples were transported to Jimma University specialized hospital using a cold box. The same day, sputum smears were examined for the presence of acid-fast bacilli (AFB) by an experienced laboratory technician using the standard Ziehl-Neelsen method [8]. Sputum samples were kept at -20°C till transported within a maximum of 5 days to Armauer Hanssen Research Institute (AHRI) in Addis Ababa for culture. At the AHRI, sputum specimens (2.5-10 ml) were processed by the standard N-Acetyl-L-Cysteine-Sodium Hydroxide (NALC-NaOH) method [9] and concentrated at 4000 × *g *for 15 minutes. The sediment, irrespective of the original sample volume, was reconstituted to 2.5 ml with phosphate buffer pH 6.8, to make the inoculums for the smears and cultures. Two Lowenstein-Jensen slants, one containing 0.75% glycerol and the other containing 0.6% pyruvate, were inoculated with the sediment and incubated at 37°C. Cultures were considered negative when no colonies were seen after 8 weeks incubation period. Isolates were harvested, Deoxyribonucleic Acid (DNA) extracted using a standardized protocol [10] and confirmed as *Mycobacterium tuberculosis *complex by an in-house Polymerase Chain Reaction (PCR) [11]. Standard spoligotyping method [12] was done generally as described by Kamerbeek and colleagues using a commercially available kit (Isogen Bioscience BV, Maarssen, the Netherlands). The SpolDB4 database [13] and a web-based computer algorithm, Spotclust http://tbinsight.cs.rpi.edu/run_spotclust.html, were used to assign new isolates to families, subfamilies and variants. SpolDB4 assigned names (shared types) were used whenever a spoligo pattern was found in the database. Patterns not found in SpolDB4 were assigned to families and subfamilies by Spotclust. Spoligotypes described only once (non-clustered) in this study and in the SpolDB4 were designated as 'NA' (not assigned). A cluster was defined as two or more isolates from different patients with identical spoligotype patterns.

### Quality control measures

As a quality control measures, we collected adequate recently-discharged mucoid or mucopurulent sputum specimen from TB suspects. The sputum specimens were transported using cold box and immediately examined for the presence of AFB by experienced laboratory technicians based on the standard procedure [8]. All the slides were checked again by the three investigators who are expert in the subject and no discrepancy was observed. Sputum samples for culture were stored at -20°C and then transported in cold boxes within five days to the reference laboratory in AHRI. Culture was done using the Lowenstein-Jensen (LJ) medium based on the standard procedure [11]. For positive samples in the culture, acid fastness was confirmed by Ziehl-Neelsen staining.

### Data analysis

Data were double entered using EpidData software version 3.1(EpidData, Norway, 2006). For analysis, the data was exported to SPSS version 15.0 statistical software (SPSS Inc. Chicago, 2007). Descriptive analysis was done to depict the socio-demographic variables and prevalence of TB. The perception of TB suspects and their health seeking behaviour is published elsewhere [14].

### Ethical consideration

The study protocol was approved by the ethical review committees of Jimma University, Institute of Tropical Medicine (ITM) in Belgium and AHRI. A workshop was held with the local community leaders, Kebele chair persons, and district health office representatives to create awareness about the purpose of the study. Written Consent was obtained from the study participants. New TB cases identified through smear microscopy and culture were immediately referred to the health facility treatment according to the national protocol.

## Results

### Characteristics of TB suspects

A total of 30040 adults in 10882 households were screened for pulmonary TB. Females constituted 50.2% of the total population. The sex composition of the survey population was similar to the whole population of Gilgel Gibe field research center. From the total adult population in ten Kebeles, 482 TB suspects gave sputum samples, however the samples collected from one Kebele were not used for culture due to delay in transportation and problems with the cold chain. Thus, a total of 428 samples (391 in rural and 91 in urban Kebeles) are presented in this paper. We managed to recruit all the TB suspects and all the TB suspects did also give adequate sputum samples. The mean age of TB suspects was 41 (SD ± 16.3); 60.7% were females; 83.6% did not had formal education; 71% were married; 90.4% were of Oromo ethnicity (Table 1).

**Table 1:** Socio-demographic characteristics of the TB suspects in Gilgel Gibe field research center, Southwest Ethiopia

Variable	No (%)
**Sex**	

Male	168(39.3)

Female	260(60.7)

**Age**, **mean(SD)**	41(16.2)

**Marital status**	

Single	50(11.7)

Married	304(71.0)

Divorced	25(5.8)

Widowed	49(11.5)

**Religion**	

Muslim	387(90.4)

Orthodox	36(8.4)

Protestant	5(1.2)

**Education**	

Had formal education	70(16.4)

No formal education	356(83.6)

The average family size of the TB suspects was 5.5 (SD ± 2.5). A total of 257 (60%) of the households of TB suspects had no window, 250 (58.4%) had no separate kitchen and 255 (59.6%) had two rooms for living. A total of 157 (36.7%) of the TB suspects were living in close contact with cattle.

More than 70% of TB suspects had fever in addition to the cough. The mean duration of cough was 13.7 weeks (SD ± 2.3); 154 (36%) TB suspects had a cough for more than 8 weeks duration.

### Prevalence of TB

Among the TB suspects, 9 (1.8%) were found to have TB based on smear microscopy. Five of them were newly identified TB cases; four were already on anti-TB treatment. Thus, the prevalence of TB using smear microscopy was 30 per 100000 populations. A total of 17 new cases of TB were identified by culture of whom 10 (59%) were females. Including the four TB patients who were on anti-TB treatment and the total population of the nine Kebeles as a denominator (n = 27597), the prevalence of TB using the culture method was 76.1 per 100,000 population. The distribution of TB suspects and TB cases detected by culture is depicted in Table 2. The ratio of passive case detection by smear microscopy in the health facilities (n = 4) vs. active case detection by culture (n = 17) was 1:4.3. This indicated that there were 4.3 undiagnosed pulmonary TB cases for every TB case who was diagnosed through the passive case detection mechanism in the health facility.

**Table 2:** Distribution of TB suspects in the study Kebeles of Gilgel Gibe Field Research Center, Southwest Ethiopia

Kebele	Total population surveyed			TB suspects			Number TB Cases identified by culture
		
	Male	Female	Total	Male	Female	Total	
Siba	1484	1409	2893	12	25	37	4(10.8%)

Asendabo	2226	2267	4493	18	23	41	0

Burka	1806	1733	3539	32	32	64	2 (3.1%)

Kejello	1662	1747	3409	16	31	47	0

Koticha	1150	1192	2342	19	24	43	0

Ayno	1704	1678	3382	17	47	64	6(9.4%)

Enkure	1015	1033	2048	16	31	47	2(4.3%)

Bore	1051	1014	2065	21	14	35	1(2.9%)

Deneba	1622	1804	3426	17	33	50	2(4.0%)

Total	**13720**	**13877**	**27597**	**193**	**260**	**428**	**17(3.9%)**

### Prevalence of HIV among TB suspects

All the TB suspects were screened for HIV and accepted the test result. All those who were rural residents were negative for HIV. Of the 91 TB suspects living in a more urban areas, 5(5.5%) were positive for HIV. Four of them were located in Deneba where there were many immigrants working at the hydroelectric power plant.

### Spoligotyping

**Figure 1:**
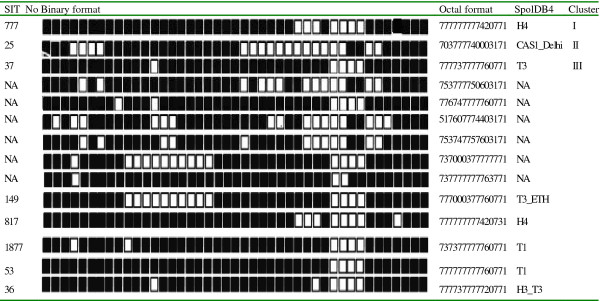
**Spoligotype pattern of *M. tuberculosis***. There were 11 isolates with distinct spoligoype pattern. NA indicates strains not found in SpolDb4 data base; Cluster represents group of two or more patient isolates that shared the same spoligoype pattern; cluster I, II and III each represent 2 isolates. Other spoligotype patterns represent single patient isolate. SIT = Spoligo-international type, CAS = Central-Asian.

A total of 14 spoligotypes were identified among the 17 *Mycobacterium tuberculosis *isolates. Six isolates (35.3%) had new and unique spoligotypes while 11 isolates (64.7%) belonged to the six previously known spoligotypes: T, Haarlem and Central-Asian. The Six isolates were grouped into three clusters (2 isolates per cluster), while the remaining 11 strains did not cluster. Of the eleven strains that did not cluster, six were new, hence represented the true orphans in the study sample. The remaining five of the unclustered isolates included strains with label T3_ETH, H4, T1 and H3-T3. One isolate lacked hybridization to spacer 33-36 and 40, which is characteristic of strains previously classified as *Mycobacterium africanum *genotype [15,16] (Figure 1).

## Discussion

Our study determined the prevalence of pulmonary TB in a predominantly rural community of Southwest Ethiopia. The prevalence of TB using smear microscopy was lower than the prevalence reported in Addis Ababa (189 per 100,000) [17], South Ethiopia (78 per 100,000) [18] and North Ethiopia (80 per 100,000) [19]. The low prevalence of HIV infection and the absence of overcrowding in this predominantly rural community could explain the low prevalence of pulmonary TB. Overcrowding and the HIV epidemic are indeed the driving forces for the spread of TB [20,21]. Using the culture method, we identified 4.3 undetected infectious TB cases for every TB patient diagnosed through the passive case detection mechanism of the health facilities. These large number of undiagnosed TB cases can transmit the disease to others particularly family members. Similar to other studies [8,22,23], we identified more undiagnosed female than male patients. Lack of empowerment to seek early health care and low knowledge of women about the cause of TB [14] could be the reasons for this high number of undiagnosed female TB patients in the community.

The spoligotyping revealed that the majority (29.4%) of the strains in our samples were of the T family, a finding which is in agreement with those of earlier studies from other parts of Ethiopia [24]. The T3-ETH was reported previously to be frequent in Ethiopia as MDR strains [25]. This clone which was characterized by low-banding IS6110-RFLP was identified in 1995 and represented 36.2% of isolates [25]. It has been described in the international database as a spoligotype that is also common in Ethiopia, Kenya and Libya [13]. The second most prevalent lineage was Harlem (H) (23.5%) which is commonly found in Poland, Saudi Arabia, Tunisia, Vietnam and Venezuela [13]. Two strains in the study sample were of the Central-Asian (CAS) family which has been described as essentially localized in the Middle East and Central Asia and preferentially in India [26].

Our study identified six new spoligotype patterns of *Mycobacterium tuberculosis *which were not present in the international database. This indicates that there is little information about *M*. *tuberculosis *strains circulating in the area. Since only a small number of isolates were characterized in our study, it is not possible to appreciate the diversity of *Mycobacterium tuberculosis *circulating in the area. More isolates and a wider geographical coverage is required to have a detail insight about the *Mycobacterium tuberculosis *strains circulating in Southwest Ethiopia.

Our study is the first of its kind in Southwest Ethiopia to assess the prevalence of TB and HIV at community level. The study tried to identify all TB suspects using repeated house to house visits. However, the study has its limitations. Fifty-four sputum samples were not examined due to an unreliable cold chain which could affect the correct estimation of the prevalence of pulmonary TB. Moreover, storage of the sputum specimens at -20°C might have an effect on the viability of *Mycobacterium tuberculosis*. This could under-estimate the culture positivity rate in our study. As a result of the small number of the isolates, a full spectrum of the *Mycobacterium tuberculosis *strains could not be documented.

In conclusion, the prevalence of TB in the rural community of Southwest Ethiopia is low. There are large numbers of undiagnosed TB cases in the community. However, the number of sputum smear-positive cases was very low and therefore the risk of transmitting the infection to others may be limited. The Ethiopian TB control program should design a strategy to improve case detection rates through the provision of tailored health education messages and involvement of lower health cadres at the grassroots level. A community-based randomized trial in Ethiopia showed that involvement of lower health cadres and health education improved the case detection rate of TB significantly (122.2% in the intervention villages vs. 69.4% in the control villages) [27]. Additional training for these lower health cadres about case detection and referral of TB cases could help the Ethiopian government to improve the case detection rate and achieve the TB related millennium development goals. We recommend researchers to conduct a large scale community-based study on the molecular epidemiology of TB to determine the strain lineage and drug sensitivity pattern in Ethiopia. A large scale study on the genotyping of *Mycobacterium tuberculosis *in Ethiopia is crucial to understand transmission dynamics, identification of drug resistant strains and design preventive strategies.

## Competing interests

The authors declare that they have no competing interests.

## Authors' contributions

AD was involved in the conception and design of the study, coordinated the field work, analyzed the data and drafted the manuscript. GA was involved in the conception, design of the study, field work and review of the article. LA was involved in the design and reviewed the article. AA, FD, KW, CJ, MT, JS, AA and MB participated in the design, field work and reviewed the article. TA did the laboratory work and reviewed the article. RC participated in the design, critically reviewed and approved the article. All authors read and approved the final manuscript.

## Acknowledgements

We acknowledge the study participants and the community for the information provided. The study was funded by Directorate General for Development Cooperation (DGDC) through the Flemish Interuniversity council (VLIR-UOS).

## Pre-publication history

The pre-publication history for this paper can be accessed here:

http://www.biomedcentral.com/1471-2334/12/54/prepub

## References

[B1] WHO Global Tuberculosis Report. WHO Report 2011 http://www.who.int/tb/publications/global_report/en/

[B2] Harries AD Dye C Tuberculosis Annals of Tropical Medicine and Parasitology 2006 5 6 415 431 16899146 10.1179/136485906X91477

[B3] Spence DP Hotchkiss J Williams CS Tuberculosis and poverty BMJ 1993 307 6907 759 761 10.1136/bmj.307.6907.759 8219945 PMC1696420

[B4] Bartlett JG Tuberculosis and HIV infection: partners in human tragedy J Infect Dis 2007 196 Suppl 1 S124 S125 17624821 10.1086/518668

[B5] WHO Global tuberculosis control: epidemiology, strategy, financing: WHO report 2009 WHO, Geneva WHO/HTM/TB/2009.411

[B6] WHO Global tuberculosis control: epidemiology, strategy, financing: WHO report 2010 WHO, Geneva WHO/HTM/TB/2009.411

[B7] Ministry of Health, Ethiopia TB, leprosy and TB/HIV prevention and control program manual, fourth edition 2007 Addis Ababa, Ethiopia http://www.moh.gov.et

[B8] International Union Against Tuberculosis and Lung Disease Sputum examination for tuberculosis by direct microscopy in low income countries 2000 5 Paris, France: IUATLD

[B9] Cruciani M Scarparo C Malena M Bosco O Serpelloni G Mengoli C Meta-analysis of BACTEC MGIT 960 and BACTEC 460, with or without solid media, for detection of mycobacteria J Clin Microbiol 2004 42 2321 2325 10.1128/JCM.42.5.2321-2325.2004 15131224 PMC404614

[B10] Van Embden JD Cave MD Crawford JT Dale JW Eisenach KD Strain identification of Mycobacterium tuberculosis by DNA fingerprinting: recommendations for a standardized methodology J Clin Microbiol 1993 31 406 409 8381814 10.1128/jcm.31.2.406-409.1993 PMC262774

[B11] Muhumuza J Asiimwe BB Kayes S Mugyenyi R Whalen C Introduction of an in-house PCR for routine identification of M. tuberculosis in a low-income country Int J Tuberc Lung Dis 2006 10 1262 1267 17131786

[B12] Kamerbeek J Schouls L Kolk A van Agterveld M van Soolingen D Simultaneous detection and strain differentiation of Mycobacterium tuberculosis for diagnosis and epidemiology J Clin Microbiol 1997 35 907 914 9157152 10.1128/jcm.35.4.907-914.1997 PMC229700

[B13] Brudey K Driscoll JR Rigouts L Prodinger WM Gori A Mycobacterium tuberculosis complex genetic diversity: mining the fourth international spoligotyping database (SpolDB4) for classification, population genetics and epidemiology BMC Microbiol 2006 6 23 10.1186/1471-2180-6-23 16519816 PMC1468417

[B14] Abebe G Deribew A Apers L Woldemichael K Shiffa J Knowledge, Health Seeking Behavior and Perceived Stigma towards Tuberculosis among Tuberculosis Suspects in a Rural Community in Southwest Ethiopia PLoS One 2010 5 10 e13339. 20948963 10.1371/journal.pone.0013339 PMC2952624

[B15] Niemann S Kubica T Bange FC Adjei O Browne EN The species Mycobacterium africanum in the light of new molecular markers J Clin Microbiol 2004 42 3958 3962 10.1128/JCM.42.9.3958-3962.2004 15364975 PMC516319

[B16] Niemann S Rusch-Gerdes S Joloba ML Whalen CC Guwatudde D Mycobacterium africanum subtype II is associated with two distinct genotypes and is a major cause of human tuberculosis in Kampala, Uganda J Clin Microbiol 2002 40 3398 3405 10.1128/JCM.40.9.3398-3405.2002 12202584 PMC130701

[B17] Demissie M Zenebere B Berhane Y Lindtjorn B A rapid survey to determine the prevalence of smear-positive tuberculosis in Addis Ababa Int J Tuberc Lung Dis 2002 6 7 580 584 12102296

[B18] Shargie B Yassin M Lindtjorn B Prevalence of smear-positve pulmonary tuberculosis in a rural district of Ethiopia Int J Tuberc Lung Dis 2006 10 1 87 92 16466043

[B19] Yimer S Holm-Hansen C Yimaldu T Bjune G Evaluating an active case finding strategy to identify smear-positive tuberculosis in rural Ethiopia Int J Tuberc Lung Dis 2010 13 11 1399 1404 19861013

[B20] Lonnroth K Jaramillo E Williams BG Dye C Raviglione M Drivers of tuberculosis epidemics: the role of risk factors and social determinants Soc Sci Med 2009 68 12 2240 2246 10.1016/j.socscimed.2009.03.041 19394122

[B21] Dye C Maher D Weil D Espinal M Raviglione M Targets for global tuberculosis control Int J Tuberc Lung Dis 2006 10 4 460 462 16602414

[B22] Subramani R Radhakrishna S Frieden T R Rapid decline in prevalence of pulmonary tuberculosis after DOTS implementation in a rural area of South India Int J Tuberc Lung Dis 2008 12 916 920 18647451

[B23] Hamid Salim MA Declercq E Van Deun A Saki KA Gender difference in tuberculosis: a prevalence survey done in Bangladesh Int J Tuberc Lung Dis 2004 8 952 957 15305476

[B24] Agonafir M Lemma E Wolde-Meskel D Goshu S Santhanam A Phenotypic and genotypic analysis of multidrug-resistant tuberculosis in Ethiopia Int J Tuberc Lung Dis 2010 14 1259 1265 20843416

[B25] Hermans PW Messadi F Guebrexabher H van Soolingen D de Haas PE Analysis of the population structure of Mycobacterium tuberculosis in Ethiopia, Tunisia, and The Netherlands: usefulness of DNA typing for global tuberculosis epidemiology J Infect Dis 1995 171 1504 1513 10.1093/infdis/171.6.1504 7769285

[B26] Bhanu NV van Soolingen D van Embden JD Dar L Pandey RM Predominace of a novel Mycobacterium tuberculosis genotype in the Delhi region of India Tuberculosis (Edinb) 2002 82 105 112 10.1054/tube.2002.0332 12356462

[B27] Datiko DG Lindtjørn B Health Extension Workers Improve Tuberculosis Case Detection and Treatment Success in Southern Ethiopia: A community Randomized Trial PLoS One 2009 4 5 e5443 10.1371/journal.pone.0005443 19424460 PMC2678194

